# Optimization of medium components for production of chitin deacetylase by *Bacillus amyloliquefaciens* Z7, using response surface methodology

**DOI:** 10.1080/13102818.2014.907659

**Published:** 2014-07-18

**Authors:** Yuanhao He, Jianping Xu, Shengjie Wang, Guoying Zhou, Junang Liu

**Affiliations:** ^a^ Key Laboratory for Non-wood Forest Cultivation and Conservation of the Ministry of Education, Central South University of Forestry and Technology, Changsha, P.R. China; ^b^ College of Forestry, Central South University of Forestry and Technology, Changsha, P.R. China

**Keywords:** chitin deacetylase, Plackett–Burman design, Box–Behnken response surface methodology

## Abstract

Plackett–Burman design and Box–Behnken response surface methodology (RSM) was employed to optimize the medium components for the chitin deacetylase (CDA) activity from *Bacillus amyloliquefaciens* Z7. Plackett–Burman design was applied to determine the specific medium components affecting CDA activity and found that starch, chitin and MgSO_4_ were critical in augmenting CDA activity. These significant parameters were further optimized using Box–Behnken RSM and the optimum concentrations of starch, chitin and MgSO_4_ were found to be 24.4, 8.8 and 0.19 g/L, respectively. The optimum medium composition was chitin 8.8 g/L, starch 24.4 g/L, yeast extract 10g/L, MgSO_4_ 0.19 g/L, K_2_HPO_4_ 0.3 g/L and NaCl 5 g/L. Under these optimal conditions, the CDA activity of *Bacillus amyloliquefaciens* Z7 increased distinctly from 18.75 to 27.48 U/mL (46.6% increase in total yield).

## Introduction

The enzyme chitin deacetylase (CDA) (EC 3.5.1.41) is a key enzyme that catalyses the conversion of chitin to chitosan by the deacetylation of N-acetyl-D-glucosamine residues. It is a member of the carbohydrate esterase family 4, according to the CAZY database.[1] Considering that CDAs play very important roles in the biological attack and defence systems, they may find applications for the biological control of fungal plant pathogens or insect pests in agriculture and for the biocontrol of opportunistic fungal human pathogens.[2] CDAs have been isolated from several fungi, e.g. *Absidia coerulea*,[3] *Aspergillus nidulans*,[4] *Colletotrichum lindemuthianum* ATCC56676,[5] *Colletotrichum lindemuthianum* DSM63144,[6] *Flammulina velutipes*,[7] *Mucor rouxii*,[8] *Rhizopus circinans*,[9] *Rhizopus nigricans*,[10] *Saccharomyces brevicaulis*,[11] and *Saccharomyces cerevisiae*.[12]

Today, chitosan is mainly produced from chitin via chemical NaOH pyrolysis, which has some disadvantages, such as poor quality of the resulting chitosan, high energy consumption and environmental pollution. These problems could theoretically be overcome by the use of CDA-producing bacteria for chitin N-deacetylation. We have previously screened and isolated a *Bacillus amyloliquefaciens* strain (Z7) with high CDA activity. The high CDA activity of this strain is one of the critical factors in favour of its application, but the optimal fermentation medium has not been studied.

Statistical methodologies are useful tools to study the interaction between the physiological factors that play important roles in biotechnological processes.[13] Response surface methodology (RSM) is considered an accurate, effective and simple approach for optimization of the experimental process [14,15] and has been successfully used in agriculture, biology, food, chemistry and other fields.[16–18]

The present study focuses on improving CDA activity of *Bacillus amyloliquefaciens* Z7. Taking CDA activity as the index, the medium components were optimized using Plackett–Burman design and Box–Behnken RSM.

## Materials and methods

### Micro-organism

*Bacillus amyloliquefaciens* Z7 was isolated from soil of the banks of the Xiangjiang River in Changsha City, Hunan, China. Strain Z7 was maintained in the Central South University of Forestry and Technology Strains Conservation Center at 4 °C and subcultured every four weeks.

### Enzyme activity assay

CDA activity was determined by two different assays. (1) Acetate released by the action of CDA on various chitinous substrates was determined by the enzymatic method of Bergmeyer [19], via three coupled enzyme reactions, as previously described in greater details.[20]

(2) A radiometric assay,[21] in which CDA activity was estimated using as substrate partially O-hydroxyethylated chitin (glycol chitin) radiolabelled in N-acetyl groups, was performed as described by Tsigos and Bouriotis.[6] The substrate was prepared according to Araki and Ito [22]. We define one unit of CDA activity as the amount of the enzyme required to produce 1 μmol of acetate per minute when incubated with hexa-N-acetylchitohexaose. Considering both sensitivity and speed, 50 °C and 15 min incubation were chosen as the standard assay conditions for CDA.

### Single-factor experiments

The basal medium was starch 20 g/L, yeast extract 10 g/L and NaCl 5 g/L, pH 6.5. The best carbon source, nitrogen source and inorganic salt were confirmed in our previous research.[20] Here, the purpose of the single-factor experiments was to determine the optimal substrate levels. To find the best chitin levels, chitin was added at different concentrations (2, 4, 6, 8, 10, 12 and 14 g/L). The pH of the medium was adjusted to 6.5 with a 0.1 mol/L HCl solution. For fermentation, 2 mL aliquots of the active seed culture were added to 100 mL of sterile production medium in 250 mL Erlenmeyer shaking flasks and then incubated at 37 °C in a rotary shaker (160 r/min) for 28 h.

### Plackett–Burman design

Plackett–Burman design is a commonly used statistical technique for medium optimization [23–27] and was used for the screening of media components that would affect the activity of CDA produced by strain Z7. According to the results from single-factor experiments, a total number of eight components (chitin, yeast extract, beef extract, glucose, starch, corn flour, KH_2_PO_4_ and MgSO_4_) were selected for this study, with each being represented at two levels, high (+1) and low (−1), as shown in Table 1. All experiments were performed in triplicates and the average CDA activity was considered as the response. The significance level of the effect of each factor was determined by Student's *t*-test. The level of significance (the *P* value) was also evaluated for each factor.

**Table 1.** Values for the Plackett–Burman design.

Variable	Component	−1 Value (g/L)	+1 Value (g/L)
X_1_	Chitin	5	10
X_2_	Yeast extract	10	15
X_3_	Beef extract	10	15
X_4_	Glucose	15	25
X_5_	Starch	15	25
X_6_	Corn flour	15	25
X_7_	KH_2_PO_4_	0.2	0.3
X_8_	MgSO_4_	0.3	0.6

### Steepest ascent design

The most important factors were found by analysing the Plackett–Burman design results. Response surface fitting is applicable to the real situation only at examining a small region.[28] That is why, an effective response surface fitting should build the best local approximations. The experimental design of steepest ascent was used to approach the best fermentation conditions. Three factors: chitin (X_1_), starch (X_5_) and MgSO_4_ (X_8_), were selected for steepest ascent design. The direction change and step size of the three factors were set on the basis of their effect size. Chitin and starch had a positive effect and their step size was 2 g/L; whereas MgSO_4_ had a negative effect and its step size was 0.05 g/L.

### Box–Behnken response surface methodology experiments

Box–Behnken RSM was employed to establish the optimum levels of the three variables: addition of starch (20–25 g/L), chitin (9–11 g/L) and MgSO_4_ (0.2–0.3 g/L), in Z7 fermentation medium. Starch (X_1_), chitin (X_2_) and MgSO_4_ (X_3_) were considered as independent factors, whereas the CDA activity was considered as the response. The experimental factors and levels are shown in Table 2. A full second-order polynomial model obtained by a multiple regression technique for two factors by using DESIGN EXPERT V8.0 was adopted to describe the response surface. The model equation obtained is as follows:(1) 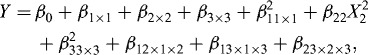

**Table 2.** Factors and levels of response surface analysis.

		Coded levels		
Independent variables	Symbol	−1	0	−1
Starch	X_1_	19.5 g/L	22.00 g/L	24.5 g/L
Chitin	X_2_	9.0 g/L	10.00 g/L	11.0 g/L
MgSO_4_	X_3_	0.3 g/L	0.25 g/L	0.2 g/L

where *Y* is the predicted response, β_0_ is the intercept, β_1_, β_2_ and β_3_ are the linear coefficients, β_11_, β_22_ and β_33_ are the squared coefficients, and β_12_, β_13_ and β_23_ are the interaction coefficients. The analyses of the data were done using DESIGN EXPERT V8.0.

## Results and discussion

### Single-factor experiments

The influence of the carbon source, nitrogen source and inorganic salt in the fermentation medium on the activity of CDA produced by *B. amyloliquefaciens* Z7 was reported in our previous research. Chitin is the substrate of CDA and, by increasing the substrate concentration, the reaction rate will increase due to the likelihood that the number of enzyme–substrate complexes will increase. The results showed that the chitin level which gave the highest CDA activity was 10 g/L (Figure S1 in the Online Supplemental Appendix). The CDA activity increased with chitin levels from 2 to 10 g/L and decreased from 10 to 14 g/L. Therefore, the most appropriate chitin levels were found to lie in the region around 10 g/L.

### Plackett–Burman experimental design

The most important three aspects of the CDA production were determined by Plackett–Burman experimental design. In Table 3, the design matrix built by the statistical software package DESIGN EXPERT V8.0 for the evaluation of eight variables in 12 experiments is presented. The coefficient and effect of each variable were calculated and the significant levels of the variables estimated by *t*-test are shown in Table 4. Among the studied variables, chitin (X_1_), starch (X_5_) and MgSO_4_ (X_8_) concentrations were significant (*P* < 0.05). Based on these results, chitin, starch and MgSO_4_ were selected as variables and applied to optimize the medium composition by RSM.

**Table 3.** Box–Behnken design of different variables with their responses.

	Variables/Levels								
Run no.	X_1_	X_2_	X_3_	X_4_	X_5_	X_6_	X_7_	X_8_	CDA activity (U/mL)
1	1	−1	1	−1	−1	−1	1	1	14.12
2	1	1	−1	1	−1	−1	−1	1	15.36
3	−1	1	1	−1	1	−1	−1	−1	19.36
4	1	−1	1	1	−1	1	−1	−1	15.98
5	1	1	−1	1	1	−1	1	−1	23.67
6	1	1	1	−1	1	1	−1	1	20.53
7	−1	1	1	1	−1	1	1	−1	13.86
8	−1	−1	1	1	1	−1	1	1	18.15
9	−1	−1	−1	1	1	1	−1	1	17.01
10	1	−1	−1	−1	1	1	1	−1	22.74
11	−1	1	−1	−1	−1	1	1	1	12.73
12	−1	−1	−1	−1	−1	−1	−1	−1	11.82

**Table 4.** ANOVA for Plackett–Burman design.

Variable	Effect	Std error	*t*	*P*-value
X_1_	3.2478	0.3242	10.0180	0.0021
X_2_	0.9485	0.3242	2.9255	0.0612
X_3_	−0.2238	0.3242	−0.6940	0.5396
X_4_	0.4535	0.3242	1.3988	0.2463
X_5_	6.2645	0.3242	19.3220	0.0003
X_6_	0.0615	0.3242	0.1897	0.8617
X_7_	0.8665	0.3242	2.6726	0.0755
X_8_	−1.5885	0.3242	−4.8995	0.0163

### Steepest ascent design

According to Plackett–Burman experiment, chitin (X_1_), starch (X_5_) and MgSO_4_ (X_8_) were selected for steepest ascent design. The direction change and step size of these three factors were set on the basis of their effect size (Table 5). The results in Table 5 show that the CDA production increased from 0 to 0 + 3Δ and decreased from 0 + 3Δ to 0 + 4Δ.. Therefore, the most appropriate central point was defined at 0 + 3Δ.

**Table 5.** Steepest ascent design and results.

Variable	Starch (g/L)	Chitin (g/L)	MgSO_4_ (g/L)	CDA activity (U/mL)
0	16	4	0.4	20.16
0 + 1Δ	18	6	0.35	22.87
0 + 2Δ	20	8	0.3	25.95
0 + 3Δ	22	10	0.25	26.79
0 + 4Δ	24	12	0.2	24.37

### Box–Behnken response surface methodology experiments

As CDA from strain Z7 was strongly affected by chitin, starch and MgSO_4_, the final medium optimization and interaction among these parameters was studied using RSM. Table 6 summarizes the response for each individual experiment along with the predicted response. The average CDA activity was taken as the independent variable or response (*Y*).

**Table 6.** Box–Behnken experiment results.

No.	Starch (X_1_)	Chitin (X_2_)	MgSO_4_ (X_3_)	CDA activity (U/mL)
1	−1	−1	0	16.34
2	−1	1	0	20.45
3	1	−1	0	28.27
4	1	1	0	23.64
5	0	−1	−1	25.14
6	0	−1	1	22.38
7	0	1	−1	24.76
8	0	1	1	21.82
9	−1	0	−1	17.83
10	1	0	−1	26.65
11	−1	0	1	18.12
12	1	0	1	24.08
13	0	0	0	26.37
14	0	0	0	26.65
15	0	0	0	26.43
16	0	0	0	26.24
17	0	0	0	25.57

Regression analysis was then performed on the obtained data. The regression equation obtained after the analysis of variance (ANOVA) gives the level of CDA produced as a function of the initial values of chitin, starch and MgSO_4_. Through regression fitting, regression equation (Equation (2)) expressed the influence of these three factors on the response:(2) 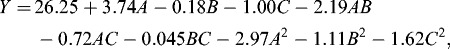
where *Y* is the response (CDA activity), and *A*, *B* and *C* are the starch, chitin and MgSO_4_ concentrations, respectively. The ANOVA analysis showed that Prob > *F* < 0.0001, indicating that the model was significant for the response (Table 7). The coefficient of determination (*R* ^2^) was calculated to be 0.9892, which ensures a satisfactory adjustment of the quadratic model to the experimental data and indicated that 98.9% of the variability in the response could be explained by the model. The adjusted coefficient of determination (Adj *R* ^2^) value of 0.9754 indicates an adequate signal. Thus, this model could be used for analysis and prediction of CDA activity.

**Table 7.** ANOVA for response surface quadratic model.

Source	Degrees of freedom (DF)	Sum of squares (SS)	Mean square (MS)	*F*-value	Prob > *F*.
Model	9	199.33	22.15	71.51	<0.0001
*A*-starch	1	111.75	111.75	360.85	<0.0001
*B*-chitin	1	0.27	0.27	0.86	0.3845
*C*-MgSO_4_	1	7.96	7.96	25.70	0.0014
*AB*	1	19.10	19.10	61.66	0.0001
*AC*	1	2.04	2.04	6.60	0.0370
*BC*	1	8.100E–003	8.100E–003	0.026	0.8761
*A^2^*	37.04	37.04	119.61	<0.0001	
*B^2^*	5.20	5.20	16.78	0.0046	
*C^2^*	11.00	11.00	35.51	0.0006	
Residual	7	2.17	0.31		
Lack of fit	3	1.50	0.50	2.99	0.1591
Pure error	4	0.67	0.17		
Cor Total	16	201.49			

^a^ *R* ^2^ = 0.9892; Adj *R* ^2^ = 0.9754.

Table 7 shows that *A*, *C*, *A* ^2^, *B* ^2^, *C* ^2^ and the interaction item of *AB* were highly significant (*P* < 0.01), and *B* and *BC* were significant at *P* < 0.5. ANOVA revealed that the three factors have an effect on the CDA activity and the interactions of *AB* and *BC* were significant. The response surfaces and contour plots in Figures S2–S7 (Online Supplemental Appendix) show that the CDA activity reached the highest level when starch was 24.4 g/L, chitin was 8.82 g/L and MgSO_4_ was 0.185 g/L. The DESIGN EXPERT V8.0 presented the maximal numerical solution with the predicted CDA activity up to 28.46 U/mL. The contour plot in Figure S3 is flat, indicating that the effects of starch and chitin on the CDA activity of strain Z7 are higher. The contour plot in Figure S5 is an ellipse, indicating that the interaction between starch and MgSO_4_ affects the CDA activity of strain Z7 more. The contour plot in Figure S7 is round, implying that the interaction between chitin and MgSO_4_ has a minimum effect on the CDA activity of strain Z7.

To confirm the model's adequacy for predicting CDA activity, a verification experiment using the optimum medium composition (chitin 8.8 g/L, starch 24.4 g/L, yeast extract 10g/L, MgSO_4_ 0.19 g/L, K_2_HPO_4_ 0.3 g/L and NaCl 5 g/L) was performed. The experiments under optimized conditions were carried out in triplicate. Under these conditions, the micro-organism produced 27.48 U/mL of CDA. The values predicted by the model were well in agreement with the results obtained for various concentrations of starch, chitin and MgSO_4_.

In previous experiment, the micro-organism produced 17.84 U/mL of CDA. Therefore, the CDA activity in this research was higher than the previous one. Considering fermentation and characterized CDA, the bacteria producing strain will be better than fungal producing strain.

## Conclusions

In this study, Plackett–Burman design, steepest ascent design and RSM were used to investigate the main and interaction effects of independent variables important for the activity of CDA produced by *B. amyloliquefaciens* Z7. The optimum medium composition for obtaining high CDA was confirmed. The significant parameters selected by Plackett–Burman screening experiments were starch, chitin and MgSO_4_. After RSM optimization, the optimized medium composition was chitin 8.8 g/L, starch 24.4 g/L, yeast extract 10 g/L, MgSO_4_ 0.19 g/L, K_2_HPO_4_ 0.3 g/L and NaCl 5 g/L; and the CDA production increased by 46.6% (from 18.75 U/mL in the unoptimized medium to 27.48 U/mL). The interaction between these components was confirmed using response surface models. Therefore, it could be concluded that the existing statistical models can successfully aid the optimization of media components for increased CDA activity.
